# An aerobe exercise intervention for optimizing metabolic, cardiovascular and immune status: protocol of an intervention study with a multi-systemic approach for women with unexplained recurrent pregnancy loss

**DOI:** 10.3389/fmed.2025.1397039

**Published:** 2025-02-13

**Authors:** Denise Habets, Aysel Gurbanova, Amber Lombardi, Salwan Al-Nasiry, Marc Spaanderman, Renate van der Molen, Lotte Wieten, Tess Meuleman

**Affiliations:** ^1^Department of Transplantation Immunology, Maastricht University Medical Centre, Maastricht, Netherlands; ^2^GROW School for Oncology and Developmental Biology, Maastricht University, Maastricht, Netherlands; ^3^Department of Laboratory Medicine, Laboratory of Medical Immunology, Radboud University Medical Center, Nijmegen, Netherlands; ^4^Department of Obstetrics and Gynecology, Maastricht University Medical Centre, Maastricht, Netherlands; ^5^Department of Obstetrics and Gynecology, Radboud University Medical Centre, Nijmegen, Netherlands

**Keywords:** recurrent pregnancy loss, immune system, cardiovascular system, metabolic system, exercise intervention

## Abstract

Women confronted with recurrent pregnancy loss (RPL) are often desperately searching for a possible explanation and hoping they will someday fulfill a healthy pregnancy. Unfortunately, in more than 50% of these women no cause for their losses can be identified after clinical investigations and therefore clinicians have no treatment options to help these women. Although adaptations in several systems such as the metabolic, the cardiovascular, and the immune system are highly important to support early pregnancy, especially the contribution of a specific subset of immune cells in the uterus known as CD56^bright^ Natural Killer (NK) cells has gained a lot of interest, investigating separate RPL associated factors might not be the way forward. Moreover, a readily available and non-invasive exercise intervention might optimize all systems simultaneously, reducing metabolic, cardiovascular and immunological risk factors contributing to RPL. Therefore, we propose an aerobe exercise intervention and study the influence on the cardiovascular, the metabolic, and the immune system, with a particular focus on endometrial CD56^bright^ NK cells, in women with unexplained RPL. In this exercise intervention study, women with unexplained RPL will receive two questionnaires to assess baseline characteristics. Moreover, they will receive (1) an immunological assessment (by sampling menstrual blood, peripheral blood and a vaginal swab) (2) an assessment of the cardiovascular system (by transvaginal ultrasound to assess uterine artery perfusion, by measuring hemodynamic and autonomic nerve system responses during a tilt test and by maximum stress test on a cycle ergometer) and (3) a metabolic assessment (by sampling peripheral blood, urine and by measuring body characteristics) before and after intervention. The intervention consists of 12-weeks moderate exercise training based on 50–65% of heart rate reserve. One year after the end of the intervention women will receive a final questionnaire regarding possible subsequent pregnancy outcome. This clinical trial with a multi-systemic approach can not only provide new insights by studying contribution and associations of the immune system, the cardiovascular system and the metabolic system in women with unexplained RPL, it also can support shared decision-making between clinicians and patients by evaluating the importance of a ready available exercise intervention strategy.

## Introduction

Recurrent Pregnancy Loss (RPL), a distressing pregnancy complication defined as two or more losses before 24 weeks of gestation, nowadays affects 1–2% of all couples trying to conceive (1). Etiologies of explained recurrent pregnancy loss can roughly be classified in embryogenic (chromosomal abnormalities in the conceptus), maternal (antiphospholipid syndrome, thyroid malfunction, anatomical uterine anomalies), and paternal (lifestyle, sperm DNA fragmentation) (1). Sadly, in at least 50% of these couples, no underlying deficit is found which leaves clinicians without means to improve their pregnancy outcome (2). It has been suggested that non-receptive adjustments of different maternal systems, such as the metabolic system (3, 4), the cardiovascular system (5, 6) and the immune system (7–9), underlie a proportion of these unexplained pregnancy losses, as all these systems need to adapt properly for early pregnancy to be successful.

During early pregnancy, the maternal metabolic system needs to undergo changes toward an anabolic state for the increasing energy demand of the developing embryo (10). In addition, the maternal cardiovascular system needs to undergo systemic hemodynamic changes and local changes in uterine artery blood flow to ensure an increasing systemic blood supply to support both the developing embryo and placenta (11, 12). As for the immune system, the composition of the uterine immune cell population is truly unique since it has to tolerate the presence of a semi-allogenic fetus, while at the same time providing sufficient immunity to infectious uterine threats (13, 14). Hereby playing a fundamental role in early pregnancy.

In the uterus, the abundance of a specific immune cell population rapidly increases in the endometrium during the mid-secretory phase of the menstrual cycle and even up to 70% in de decidua during early pregnancy (15). This population of uterine Natural Killer (NK) cells is characterized by high levels of CD56 thereby also called CD56^bright^ NK cells, in contrast to the NK cell population in peripheral blood that mainly consists of CD56^dim^ NK cells (16). It is commonly thought that these CD56^bright^ uterine NK cells have an immunomodulatory function with the ability to secrete anti-inflammatory and growth factors, hereby playing a crucial role in embryo implantation by regulating adequate trophoblast invasion and remodeling of spiral arteries in order to establish proper early placentation (17). Early pregnancy safe harvesting of human CD56^bright^ uterine NK cells without endangering ongoing pregnancy is hardly feasible, however in non-pregnant endometrium it is possible to obtain samples through biopsies. Nevertheless, biopsies from endometrial tissue are invasive and painful, limiting their use for research purposes. Instead of biopsies, endometrial NK (eNK) cells can be easily isolated from menstrual blood and have shown to have the same phenotype as cells isolated from biopsies even allowing for collection of ∼10 times more lymphocytes than biopsies (18, 19).

As the metabolic, the cardiovascular and the immune system seemingly play a major role in sustaining successful pregnancy, research in these various systems has gained interest for pregnancy complications such as RPL. Women with RPL have been shown to have an increased incidence of metabolic disorders such as hyperhomocysteinemia, altered amino acid metabolism and an increased risk for developing the metabolic syndrome (3, 20, 21). Moreover, in women with RPL, several studies have found cardiovascular aberrations (22), amongst reduced circulatory plasma volume, reduced uteroplacental perfusion, increased total peripheral resistance, and hypertension (5, 23–25), also increasing their risk for cardiovascular diseases later in life (26, 27). In addition, alterations in uterine NK cell function and phenotype in patients with RPL have also been observed. Lower numbers of the CD56^bright^ NK cell subset and increased numbers of the CD56^dim^ NK cell subset with high expression of the activating CD16 receptor were found in the endometrium and decidua of women with RPL compared to controls without a history of RPL (9, 28–30). Furthermore, enhanced secretion of the pro-inflammatory cytokines IFN-γ and TNF-α by uterine NK cells was found in women with RPL compared to women with a previous normal pregnancy (31).

Although these prior studies emphasize the contribution of the cardiovascular, metabolic and immune system in relation to RPL, no study to date has looked at the added value of interplay between these different systems. Even taking into consideration the importance of a common regulator of these various systems; the sympathetic nervous system (32). Sympathetic hyperactivity has been linked to both cardiovascular and metabolic disorders and sympathetic dysfunction has been a significant contributor to the pathophysiology of immune mediated inflammatory diseases (33–35). Valuable work to develop effective therapeutic strategies for reversal of pathological sympathetic hyperactivity (36) is ongoing, nevertheless emerging evidence from recent animal and human studies seems to highlight the importance of exercise training as a widely prescribed non-pharmacological therapeutic strategy (37–39).

Nowadays, it is becoming generally accepted that exercise is associated with beneficial effects on the cardiovascular and metabolic system and even short workouts modulate immune responses that may have clinical impact (40). More notably, exercise is known to promote healthy reproduction by reducing the risk on pregnancy complications such as preeclampsia and gestational diabetes (41). For women with RPL specifically, inconclusive effects have been found and to date no studies investigate the impact of exercise on the chance of a live birth for RPL women (1, 41). Moreover, the effect of exercise on CD56^bright^ eNK cells has never been studied although it has been found that NK cells in the periphery are the most responsive immune cells to exercise, displaying an activation and acute mobilization to the circulation during physical exertion most pronounced during moderate training (42–44).

As a low number or altered function of CD56^bright^ eNK cells, in addition to the presence of metabolic and cardiovascular risk factors might contribute to RPL, we aim to gain new insights in the link between metabolic, cardiovascular and immune status of women with unexplained RPL with a unique multi-systemic approach and try to improve their overall health status with a moderate aerobe exercise intervention.

## Methods and analysis

### Study objectives and design

The aim of this multi-center interventional cohort study is to explore differences in immune parameters, in particular in CD56^bright^ eNK cells, and in cardiovascular, and metabolic parameters in women with RPL before and after an exercise intervention. Women referred to the department of Obstetrics and Gynecology of the Radboud University Medical Centre (RUMC) and the Maastricht University Medical Centre+ (MUMC+) for RPL will be included when no identifiable cause for their losses can be determined by clinical investigations.

According to current (inter)national guidelines of the European Society of Human Reproduction and Embryology (ESHRE), the clinical work-up includes; a standardized history from the couple, maternal screening for antiphospholipid antibodies (anticardiolipin, lupus anticoagulant and β2 glycoprotein I), maternal thyroid screening, assessment of anatomical uterine anomalies, paternal screening for sperm DNA fragmentation, and parental karyotyping if indicated (1).

After obtaining written informed consent, a questionnaire to assess general baseline characteristics and a short questionnaire to assess short health-enhancing physical activity (SQUASH) (45) will be conducted. Subsequently, peripheral blood and menstrual blood will be collected for assessment of the immune system and a vaginal swab will be collected to additionally evaluate the vaginal microbiome as the microbiome is known to play a fundamental role on the induction, training, and function of the immune system (46). Assessment of the cardiovascular system will be conducted by transvaginal ultrasound of both uterine arteries and by measuring hemodynamic responses during tilt test to determine hemodynamic parameters and sympathetic nervous system activity and responsiveness. Additionally, a maximum stress test on a cycle ergometer will be performed to determine the maximum rate of oxygen (VO_2_max) the subject’s body is able to use during exercise. Metabolic status will be assessed by measuring metabolic components in peripheral blood, urine and by measurements of body characteristics.

The intervention consists of a personalized 12-week heartrate-controlled cycle training program, that must be carried out on top of a subjects regular exercise regime and diet. Heart rate will be monitored by a Polar H10 heartrate monitor chest strap during cycle trainings. If women don’t want to participate in the training intervention, they will be asked to participate in a timeline-control group in which they receive similar questionnaires and assessments of their immune, cardiovascular and metabolic system. However, instead of an additional cycle training program of 12-weeks, they only follow their regular exercise regime and diet during an identical period. In this latter group, women will be included under similar criteria as the intervention group, preferably in a 1:1 ratio. Comparability between groups will be assessed on baseline characteristics prior to the intervention.

**FIGURE 1:**
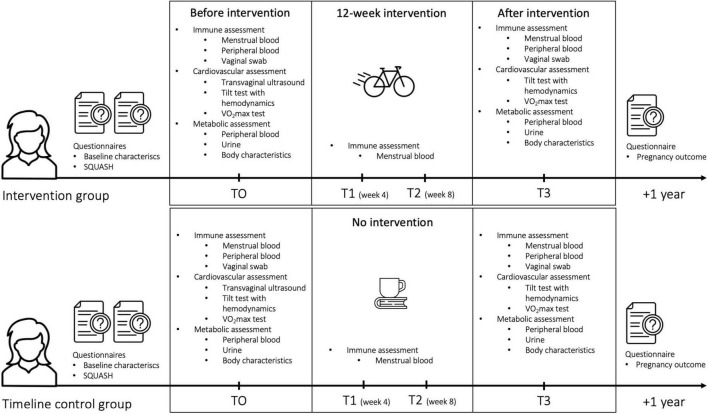
Schematic overview of study design.

For both the intervention and control group, women will be asked to additionally donate menstrual blood at 4 and 8 weeks during the 12-week interval in order to assess intra menstrual cycle variation of eNK cells. Subsequently, all women will receive an identical immune, cardiovascular and metabolic assessment after 12 weeks and 1 year after intervention, a short questionnaire for monitoring subsequent pregnancy outcomes. See Figure 1 for a schematic overview of study design.

#### Primary objective

To determine the changes in CD56^bright^ eNK cell number, phenotype and function before and after a moderate aerobe exercise intervention in women with unexplained RPL.

#### Secondary objectives

• To study baseline characteristics and baseline levels of physical activity in women with unexplained RPL.
• To study immunological effects of an aerobe exercise intervention in women with unexplained RPL and compare these between endometrial and peripheral samples.
• To compare immunological effects in women who undergo an aerobe exercise intervention and a timeline control group who do not follow an aerobe exercise intervention.
• To evaluate additional effects of an aerobe exercise intervention on the vaginal microbiota (in relationship to eNK cell population) as it can influence the maternal immune system.
• To evaluate additional effects of an aerobe exercise intervention on markers of cardiovascular and metabolic health that are known to be associated with adverse pregnancy outcomes in women with unexplained RPL.
• To study additional effects of an aerobe exercise intervention on sympathetic nervous system activity, as the sympathetic autonomic system appears to be an important modulator of the immune system, the cardiovascular system, and the metabolic system.
• To study additional effects of an aerobe exercise intervention on physical fitness levels measured by maximal oxygen consumption.
• To monitor pregnancy outcome up to 1 year after an aerobe exercise intervention.
• To investigate correlations between the immunological, cardiovascular and metabolic effects of an aerobe exercise intervention in women with unexplained RPL.

### Sample size

The effect of this interventional cohort study on CD56^bright^ eNK cells is hard to predict due to the novelty of this study using uterine NK cells as our primary objective. As for peripheral NK cells, a large meta-analysis showed an overall striking increase in cell count during moderate exercise training (186–344% of initial resting counts) (44). Since it is believed that training evokes redistribution of cells between blood and tissues due to increased hemodynamics (47), we hypothesize that moderate exercise possibly enables mobilization of NK cells to the endometrium and therefore affects CD56^bright^ eNK cell numbers. Moreover, after moderate exercise training, numbers of peripheral NK cells overall decreased to their initial NK cell count along with an analogous depression of cytolytic activity before complete recovery (44). Although at first these changes per training seem unlikely to have a major influence on general population health, it could be that a cumulative period of immune depression becomes of greater importance, especially to reproductive health. As these studies from this meta-analysis included around 20 subjects to show an effect on peripheral NK cells, we will aim to end up with around 20 women for data analysis. Furthermore, based on previous research showing discriminatory levels of the peripheral NK cell marker LILRB1 in two independent studygroups (8) we expect a detectable significant difference for a mean fluorescent intensity of 473 between group 1 (mean 771 ± 600) and group 2 (mean 298). When comparing two independent study groups for a continuous endpoint with an alpha of 0.05 and a beta of 0.2, a total number of 50 women, 25 in each group is needed to detect this difference of 600 and show discriminatory levels of NK cell markers. Additionally, we performed a pilot study to evaluate the feasibility of training women with RPL, to determine the modifiability of risk factors for RPL (evaluations of CD56^bright^ eNK cells were not part of this pilot study). We found that already after 1 month of supervised training, 9 out of 12 women significantly showed an improved VO_2_max (26.7 versus 27.4, *p* = 0.024) and one woman was unable to complete her trainings (unpublished data). Therefore, we expect that after a longer period of physical training at least 75% of the included women will improve their physical fitness (48).

In conclusion, we expect a clinically significant training-induced improvement with the inclusion of 30 women, assuming a similar effect on eNK cells as in peripheral blood. This allows for a drop-out rate of approximately 15%, leaving around 25 subjects for analysis hereby considering a compliance of 75% to the training intervention.

### Study population and recruitment

Eligible women with two or more unexplained pregnancy losses, will be included during a visit at the RPL clinic at MUMC+ or first trimester pregnancy clinic at the RUMC. These women are mostly referred by their general practitioner or other regional clinics in the southern or eastern part of the Netherlands. A pregnancy prior to a pregnancy loss, must be confirmed at least by either serum or urine beta-human chorionic gonadotropin, as ectopic and molar pregnancies are excluded in this definition. According to current (inter)national guidelines, the clinical work-up of women with RPL includes; a standardized history from the couple, maternal screening for antiphospholipid antibodies (anticardiolipin, lupus anticoagulant and β2 glycoprotein I), maternal thyroid screening, assessment of anatomical uterine anomalies, paternal screening for sperm DNA fragmentation, and parental karyotyping if indicated (1). Only women with unexplained RPL, not aiming to conceive within the study time frame of 3 to 4 months are eligible to participate. Furthermore, to ensure that there is no influence of a recent pregnancy loss or birth, women should be at least 2–3 months after their last pregnancy loss or birth. Further exclusion criteria are age above 40 years, body mass index above 40, current use of immune suppressive medication or hormonal contraceptives, current infection with the human immunodeficiency virus or symptomatic genital infections such as chlamydia or gonorrhea, pre-existent diabetes mellitus, autoimmune disease or overt cardiovascular disease, and vaccination 1 month prior to or during sampling and intervention.

On average, 30 new couples who experienced RPL visit our clinic every month, of which approximately 15 women have unexplained RPL. These women will be informed about the study by the health professionals in our clinic and a patient information folder will be handed out to women who are interested to read about the study in more detail. The health professional also asks for permission to pass their contact information to the study investigator. Within 1 week women will be contacted by the study investigator to get additional verbal information about the study and answer possible questions. During this consultation, the study investigator will ensure that all subjects will be given full and adequate verbal and written information about the nature, purpose, possible burdens and benefits of the study. Furthermore, it will be emphasized that participants are free to withdraw from the study at any time without specific reason. After eligibility is verified, women are free to take the time they need before deciding if they would like to participate or not. We estimate that monthly approximately 4 out of these 15 eligible women would like to participate in the intervention study. Additionally, we anticipate extra participants via RiNet (Reproductive Immunology network in the Netherlands)^1^ and via Freya (Dutch association for women with fertility problems).^2^ Information about the study and contact details will be provided on both websites. In this way, women who are interested in possible participation can contact the study investigator directly. Further course will be the same as for women who are informed about the study in the RPL clinic at MUMC+ or first trimester pregnancy clinic at the RUMC.

In our clinical experience we observed profound willingness in this specific patient group to participate in scientific studies which potentially help them and future patients. Our inclusion rate is however anticipated on the more modest side, because we do respect practical concerns, mainly related to time investment issues in couples who would like to conceive on short notice.

A total of 30 women with unexplained RPL will be included in this study to participate in the intervention group, additionally a maximum of 30 RPL women will be included under similar criteria in the timeline control group. Allocation was non-randomized as patients were allocated based on what was considered most appropriate for their individual circumstances.

### Study procedures and data collection

In short, a questionnaire on baseline characteristics and on baseline exercise levels will be conducted before intervention. For immunological assessment, menstrual blood will be sampled before, during, and after intervention. Peripheral blood and a vaginal swab will be sampled before and after intervention. For assessment of the cardiovascular system, a transvaginal ultrasound examination of both uterine arteries will be performed before intervention. Hemodynamic responses during a tilt test and maximal oxygen consumption during a VO_2_max test will be measured both before and after intervention. For metabolic assessment, measurements of metabolic components in peripheral blood and urine plus body characteristics will be determined before and after intervention. The intervention consists of 12-weeks moderate training. One year after intervention women will receive a final short questionnaire regarding pregnancy outcome.

#### Questionnaires

Before the intervention women will receive two questionnaires; one for assessing baseline characteristics such as age, medication use, information on gynecological history and obstetric history and one for standardized assessing baseline exercise levels. A year after completion of the intervention, women will receive a short digital questionnaire with questions about subsequent pregnancy outcome.

#### Immune assessment

• Menstrual blood and peripheral blood

Peripheral blood will be collected during a visit to the RUMC or MUMC+. Menstrual blood will be self-collected by the subject during 4 cycles before (T0), during (T1: week 4 in intervention, T2: week 8 in intervention) and after the intervention (T3). Women will receive verbal and written instructions on how to collect menstrual blood. Each cycle menstrual blood will be collected in 3 blocks of 12 h (total of 36 h) by use of a menstrual cup. A menstrual cup is a small silicon cup that can be used similarly like a tampon. All materials necessary to collect menstrual blood, including one menstrual cup since this cup can be re-used, will be send to the participant by postal service. When collection of the samples is completed, the material will be delivered in the hospital or sent to the lab directly by postal service. Our study group has gained experience with this protocol in previous studies (9, 18).

Peripheral blood mononuclear cells (PBMC) and menstrual mononuclear cells (MMC) will be isolated from the peripheral and menstrual blood by density gradient centrifugation. Number, phenotype, and function of these cells will be studied using flow cytometry. In short, NK cell receptors related to activation, inhibition, and memory function, will be analyzed. Functionality of NK cells will be determined by measuring the intracellular production of cytokines. Furthermore, cytotoxic potential of NK cells will be investigated by culturing NK cells with K562 target cells and degranulation potential will be assessed by CD107 expression and cytokine analysis such as IFN-γ production of different NK cell subsets. Additionally, menstrual mononuclear cells will be used for metabolomic analyses, targeting several lipids including glycerophospholipid and sphingolipid metabolism and microbiome related metabolites.

• Vaginal swab

Vaginal swabs for microbiome analyses will be collected with Copan flocked swabs and placed in eNAT buffer and stored at −20°C. DNA extraction of the vaginal swabs will be performed using a buccal swab extraction kit. Briefly, collected swabs samples will be thawed, vortexed and incubated with lysis buffer, proteinase K and elution buffer. For interspace profiling, amplification of the intergenic spaces (IS) regions will be performed with the IS-pro assay. IS-pro is a eubacterial technique based on the detection and categorisation of the length of the 16S-23S rRNA gene IS region, as the length of this IS region is specific for each bacterial species. Phylum-specific fluorescently labeled PCR primers will be used for taxonomic classification.

#### Cardiovascular assessment

• Transvaginal ultrasound

To evaluate blood flow to the uterus, the resistive index (RI) and pulsatility index (PI) of both uterine arteries will be examined by transvaginal route. After obtaining a sagittal image of the uterus, cervical canal and detection of the internal ostium, the uterine artery will be identified via color flow mapping and will be measured by pulsed-wave Doppler flow velocity waveforms of the ascending branch of both uterine arteries.

• Hemodynamic responses during tilt test

A tilt test will be performed under standardized environmental conditions by an experienced researcher. During the tilt test a woman first remains supine for 20 min, after which her body posture will passively be changed from 20° head-down tilt (−20°) to 60° head-up tilt (+60°), in steps of 20° at 8-min intervals. Heartrate and arterial blood pressure will be measured continuously using a monitoring device, called a Finometer, attached to a finger. The activity of the sympathetic nervous system will be derived by means of an arithmetic processing of the beat-to-beat changes in heartrate and blood pressure over time (47).

• VO_2_max test

Physical fitness will be determined as the peak oxygen uptake (VO_2_max: milliliter minute^–1^ kilogram^–1^) during a maximal cycling test on a cycle ergometer. The VO_2_max test will be performed under standardized environmental conditions and by an experienced researcher. During the test several parameters will be measured continuously. These non-invasive measurements include breath oxygen uptake by means of analysis of inhalation (O_2_ input) of and exhalation (CO_2_ output) and heartrate plus rhythm by electrocardiogram. The VO_2_max test will endure cycling in total for 10 to 30 min, with every minute an increase of 10 watts until total exhaustion.

#### Metabolic assessment

• Peripheral blood and urine

Collection of peripheral blood will be done during a visit to the RUMC or MUMC+ at a similar timepoint as for the immune assessment so that women need to donate blood only once. A first-morning urine sample will be self-collected. Blood and urine samples will be sent to the central diagnostics laboratory of the RUMC or MUMC+ for standardized assessment of metabolic components such as triglycerides and cholesterol levels, glucose, insulin, and creatinine.

• Body characteristics

Measurements of body characteristics such as weight, height and waist-hip circumference will be performed.

#### Intervention

Our moderate exercise intervention will consist of a 12-week heartrate-controlled training program for a cycle trainer. Trainings will take place at the Radboud Sportcentrum in Nijmegen, at the UM Sports in Maastricht or in exceptional cases at a local sports facility of choice.

During the intervention, the subjects will be asked to follow the same diet as prior to study inclusion and are instructed not to participate in novel exercise regimes in addition to the training program that they were given.

The training program is personalized to heartrate reserve (HHR) and can be calculated in the following manner: HHR = HR_max_ minus HR_rest_, in which HR_max_ is the maximal heartrate measured during the VO_2_max test before intervention and HR_rest_ is the heartrate determined at rest. The program consists of 60 min of cycling for 2 times (week: 1–6) to 3 times (week: 7–12) per week. Each training will start with a warming-up of 5 min on 40% of the individual HHR, followed by a training intensity of 50 min on 50–65% of HRR and a cooling down of 5 min on 40% HRR. For convenience, heartrate zones according the HRR will be predetermined. During each training heartrate can be monitored continuously and recorded by a Polar chest band and results can be viewed on the subject’s personal Polar account.

Each week, the subject will be asked to send results of the trainings to the researcher so read out of heartrates can be evaluated to ensure adequate adherence to the training protocol. During weekly consultations by phone, the researcher will monitor compliance and motivate subjects to complete the intervention.

### Statistical analysis

Normality of data will be evaluated with Kolmogorov-Smirnov tests or visual inspection of histograms. Descriptive data will be expressed as mean ± standard deviation for parametric continuous variables or as median and interquartile range for non-parametric continuous parameters or percentage for categorical variables. To test individual effects of our exercise intervention, parameters of the immune, metabolic, or cardiovascular assessments respectively, will be compared before versus after intervention, for which data will be assessed by parametric paired T-tests or non-parametric Wilcoxon signed rank tests. Moreover, to test group effects of our exercise intervention, parameters of the respective systems will be compared between the intervention and the time control group, for which data will be compared by parametric T-tests or non-parametric Mann Whitney U tests. Since we assume that the different systems might influence each other in the occurrence of recurrent pregnancy loss, correlation analyses will be used to examine interrelationships, specifically correlating changes of NK cell phenotypes with metabolic and cardiovascular parameters. Correlations between immune, metabolic and cardiovascular data will be assessed by univariate and multivariate logistic regression analysisand calculated as crude and adjusted odds ratios to correct for possible confounders such as BMI. Statistical significance will be defined as *P* < 0.05 using SPSS version 28.0.

### Data management

Data will be collected in a single electronic cloud-based clinical data management platform; Castor EDC by the coordinating investigators of the RUMC and MUMC+. Furthermore, coordinating investigators will maintain adequate study records, including laboratory forms, signed informed consents, and will ensure that the subject’s anonymity will be guaranteed by confidentially coding patient data. In all documents, subjects will be identified by an identification code only and thus not by their names or any other feature by which subjects can be identified. The investigator will keep a separate Subject Identification Code List stored at the Department of Laboratory Medicine, Laboratory of Medical Immunology RUMC and Department of Transplantation Immunology MUMC+, which matches identifying codes with the subjects’ names. Therefore, only coded samples will be handled in the laboratory and the information is not directly traceable to the patient. The Code List will be maintained by the investigator in strict confidence. Access will be granted only to members of the research team if necessary, and to the study monitor if requested. Subject anonymity will be maintained throughout all archiving steps. All samples will be coded as followed: RUM-001 or MUM-001 dependent if sampling takes place in Nijmegen or Maastricht. All data will be handled confidentially.

All data will be stored and archived through the data management infrastructure of the institute, which will be kept for at least 15 years (following guidelines for minimal risk research Kwaliteitsborging Mensgebonden Onderzoek 2.0 + institutional DR policy). Materials will be stored for further investigation concerning this protocol with their corresponding code at −20°C, −80°C or within a liquid nitrogen storage for a maximum of 10 years. Following publication, raw and processed data will be archived for scientific integrity, which is only accessible upon request through the coordinating investigators and the researchers responsible for the data.

### Adverse events

All adverse events (AE) and serious adverse events (SAE) reported spontaneously by the subject or observed by the coordinating investigator (or his staff) will be recorded without undue delay after obtaining knowledge of the events. However, we expect the number of (S)AE’s during the study to be minimal. As the training will be performed on a stationary cycling trainer and training will only be on 50–66% of HRR, the risk on a heavy sport injury is expected to be negligible. We anticipate some mild muscle ache in the first weeks of the training protocol, especially in previously sedentary women. We try to minimize any sport related risks and injuries by starting with cycling training two times a week in the first 6 weeks and increase after 6 weeks to 3 times a week to allow a more gradual increase in physical fitness. Additionally, each training will start with a warming-up of 5 min and end with a cooling down of 5 min. Training will take place in a center with routine surveillance of sport instructors, which could be consulted in case of injury. Participants will be actively asked to report any soreness, injuries or adverse effects during the weekly support session with the researcher. When applicable, the principal investigator will report all (S)AEs to the sponsor without undue delay after obtaining knowledge of the events. Recorded (S)AEs will be followed up by the coordinating investigator who maintains regular telephone contact with the participant until (S)AEs have abated, or until a stable situation has been reached. Depending on the event, follow-up may also require referral to a general physician or medical specialist. Verification of the reports on adverse events and complications will be carried out by a study monitor of the RUMC.

### Monitoring

A certified independent party of the RUMC will monitor the study in both the RUMC as the MUMC+ according to the monitor plan. A first visit was planned before start of the study, a second visit was planned after inclusion of the first 5 subjects. Interim assessment is not planned yet. Safety surveillance by a safety monitoring board is not implemented given the low risk of adverse events.

### Patient and public involvement

Information and results of the study will therefore be shared on a national patient platform for women with reproductive infertility (see text footnote 2) and on a personalized website that will temporarily be active.

## Discussion

RPL is one of the most complex and challenging scenarios in reproductive medicine. Understanding the mechanisms behind RPL and identifying mediators or effectors and validating targets for prevention or therapy will have a profound impact on the couples’ decision making on future family planning. However, identifying one single causal factor is challenging to say the least. A multidisciplinary approach seems to be the way forward and can be of great value for identifying a subset of women with RPL with an underlying high-risk etiology. To date, there are no multi-systemic diagnostic or intervention strategies available for women with RPL. However, since there is a limited availability of predictors and possibilities for diagnosis and treatment of RPL, it is important to investigate novel factors that can offer more perspective for these women. Therefore, this intervention study with a multi-systemic approach could provide new insights by studying contribution and associations of the immune system, the cardiovascular system and the metabolic system in women with unexplained RPL. Moreover, there is great novelty in investigating the effect of an exercise intervention on pregnancy supportive immune cells in the uterus, including endometrial CD56^bright^ Natural Killer cells. Although prediction of a healthy pregnancy following intervention will be difficult due to small sample size, results of this explorative clinical trial could provide valuable evidence for subsequent trials with larger cohorts, hereby making it possible to study RPL women in high- versus low-risk subgroups. Furthermore, results of this trial can be shared with the scientific community and with patient platforms to provide more insight in RPL related mechanisms and to support shared decision-making between clinicians and patients in order to select the most suitable treatment option based on both best available evidence and informed patient preferences.

## Footnotes

1
www.RiNet.nl

2
www.freya.nl
